# Supplementary material for: Deletion of wfs1 Impairs Oligodendrocyte Precursor Cells Dorsal Distribution and Myelination Through the wfs1-hmgcs1 Axis in Zebrafish
Source: Biology (Basel). 2026 Aug 16;15(16):1402. doi: 10.3390/biology15161402 (PMC13510151; doi:10.3390/biology15161402)
Supplement: Supplementary file 1 [file biology-15-01402-s001.zip › File S1-Transcriptome sequencing data and differentially expressed genes (DEGs) in wfs1-- zebrafish at 5 dpf/2.KEGG/wfs1KOvsWT/ALL/src/dre04020.html]

dre04020


- dre:368717

- Up regulated genes

368717(4.12704824157962)

- Down regulated genes

368717\_1(-1.74128930765284)

- dre:393186

- Up regulated genes

393186\_1(3.79579966743607)

- dre:565241

- Up regulated genes

565241(1.32977867526604)

- Down regulated genes

565241\_2(-6.09874532631292)

- dre:100006675

- Up regulated genes

100006675(1.63301233543285)
- dre:497384

- Down regulated genes

497384\_1(-2.05267237809407)

- dre:101886891

- Up regulated genes

101886891(1.44936351432932)

- dre:544664

- Down regulated genes

544664(-5.14896748950436)
- dre:386856

- Down regulated genes

386856\_1(-1.99487147218035) novel.946(-6.7676180476835)
- dre:100149258

- Up regulated genes

544664\_1(3.28411269336583)
- dre:569566

- Down regulated genes

569566(-1.00953785382545)
- dre:571431

- Down regulated genes

571431(-6.42862549702529)
- dre:796537

- Down regulated genes

796537\_1(-2.50164719187441)

- dre:387296

- Down regulated genes

387296\_1(-5.24544706670971)
- dre:58147

- Up regulated genes

58147(4.28011810579641)

- Down regulated genes

58147\_1(-3.00265513562539)
- dre:259258

- Down regulated genes

259258(-3.56445279283737)
- dre:570493

- Down regulated genes

570493\_1(-1.68806351340199)

- dre:569706

- Up regulated genes

569706\_1(4.25727678218935)

- dre:566671

- Down regulated genes

566671(-1.07930365706202)

- dre:555941

- Up regulated genes

555941(1.09497190188701)

- dre:492486

- Up regulated genes

492486(4.56703139609518)

- Down regulated genes

492486\_1(-1.65362606223537)
- dre:100000981

- Down regulated genes

100000981\_2(-1.61456337824489)
- dre:794365

- Up regulated genes

794365(1.73411710545423)
- dre:559806

- Up regulated genes

559806(2.30033119315707)
- dre:100137117

- Up regulated genes

100137117\_1(2.43790601872866)

- Down regulated genes

100137117(-1.33851850264817)

- dre:192322

- Up regulated genes

336121(1.42904963424916)

- dre:353247

- Down regulated genes

353247(-1.16019504230133)

- dre:568726

- Up regulated genes

568726(1.14419230813088)

- dre:100005590

- Up regulated genes

100005590(4.26358459183019)

- Down regulated genes

100005590\_1(-4.43307927362236)

- dre:558525

- Up regulated genes

558525\_1(3.69011072176884)

- dre:445226

- Up regulated genes

445226(6.27633199587492)

- dre:100005049

- Down regulated genes

100005049(-1.30748784788409)
- dre:560564

- Down regulated genes

560564\_2(-4.02056550705436)
- dre:796490

- Up regulated genes

796490\_2(1.58582846768856)
- dre:494456

- Up regulated genes

494456(1.65469795112832)
- dre:103909601

- Up regulated genes

103909601(4.16535952448207)

- Down regulated genes

378480(-1.33145438810737)
- dre:58133

- Up regulated genes

58133(1.32041761049897)

Close
